# Relatively preserved functional immune capacity with standard COVID-19 vaccine regimen in people living with HIV

**DOI:** 10.3389/fimmu.2023.1204314

**Published:** 2023-09-04

**Authors:** Chen-Yiu Hung, Sung-Han Hsiao, Chung-Guei Huang, Chia-Shiang Chang, Guan-Yan Chen, Yu-Lin Huang, Avijit Dutta, Ching-Tai Huang

**Affiliations:** ^1^ Department of Thoracic Medicine, Chang Gung Memorial Hospital, Taoyuan, Taiwan; ^2^ Division of Infectious Diseases, Department of Medicine, Chang Gung Memorial Hospital, Taoyuan, Taiwan; ^3^ Department of Laboratory Medicine, Chang Gung Memorial Hospital, Taoyuan, Taiwan; ^4^ Department of Medical Biotechnology and Laboratory Science, College of Medicine, Chang Gung University, Taoyuan, Taiwan; ^5^ Research Center for Emerging Viral Infections, College of Medicine, Chang Gung University, Taoyuan, Taiwan; ^6^ Department of Infectious Diseases, College of Medicine, Chang Gung University, Taoyuan, Taiwan

**Keywords:** PLWH, COVID-19 vaccine, standard 2-shot regimen, an additional shot of primary series COVID-19 vaccine, virus neutralizing antibody, antigen-specific T cell response

## Abstract

**Introduction:**

People living with HIV (PLWH) are at a higher risk of severe disease with SARS-CoV-2 virus infection. COVID-19 vaccines are effective in most PLWH. However, suboptimal immune responses to the standard two-shot regimen are a concern, especially for those with moderate to severe immunodeficiency. An additional dose is recommended as part of the extended primary series in Taiwan. Herein, we study the efficacy of this additional shot in PLWH with mild immunodeficiency compared to that in healthy non-HIV people.

**Methods:**

In total, 72 PLWH that were asymptomatic or with mild immunodeficiency (CD4 counts ≥200/mm^3^) and suppressed virology, and 362 healthcare workers of our hospital were enrolled. None of the participants had a history of SARS-CoV-2 infection. They received mRNA-1273 and ChAdOx1 vaccines. Anti-SARS-CoV-2 neutralizing and anti-Spike IgG antibodies, and SARS-CoV-2-specific T cell responses were evaluated.

**Results:**

The standard two-shot regimen elicited lower responses in PLWH than the healthcare workers without HIV infection, although the difference was statistically insignificant. They had comparable levels of neutralizing and anti-Spike antibodies and comparable effector CD4+ and CD8+ T cell responses. The third shot boosted the SARS-CoV-2 immunity significantly more with better antibody responses and higher IFN-γ and IL-2 responses of the CD4+ and CD8+ T cells in PLWH compared to those without HIV. Upon *in vitro* stimulation with extracted Wuhan strain SARS-CoV-2 proteins, CD8+ T cells from PLWH after 3 shots had more durable effector responses than the non-HIV controls with extended time of stimulation.

**Conclusion:**

This subtle difference between PLWH and non-HIV people implied immune exhaustion with two shots in non-HIV people. Slightly compromised immunity in PLWH indeed preserved the functional capacity for further response to the third shot or natural infection.

## 1 Introduction

Vaccination is an effective measure to counteract infectious diseases. It is also of critical value in the coronavirus disease-19 (COVID-19) pandemic (1, 2). However, most COVID-19 vaccine studies did not address immunocompromised individuals and the data on vaccine efficacy in people living with HIV (PLWH) are limited. As PLWH have a compromised immune system (3), they are more vulnerable to the SARS-CoV-2 infection and are presumed to respond less to vaccines (4, 5). Furthermore, antiretroviral therapy (ART) for HIV increases the incidences of comorbidities such as diabetes, cardiovascular diseases, and kidney diseases in PLWH with time and age (6, 7). People with comorbidities are prone to SARS-CoV-2 infection with increased mortality (8, 9). It is then a concern whether the standard two-shot regimen of COVID-19 vaccination can confer sufficient immunity in PLWH (10–12).

ART suppresses the virus and improves immune function to turn the disease into a chronic condition with high life expectancy in PLWH (13). With appropriate ART, the majority of PLWH have a CD4+ T cell count ≥ 200/mm^3^ and no detectable virus (14). COVID-19 vaccines are effective in these people and perhaps reduce the risk of severe disease or death with the standard two-shot regimen (15). However, suboptimal immune response due to relative immune deficiency to the standard regimen is still a concern (16, 17). An additional dose may be recommended as part of the extended primary series. In Taiwan, this additional dose is endorsed even for those who are asymptomatic or have mild immunodeficiency with CD4 counts ≥ 200/mm^3^ and suppressed virus load. We aimed to investigate the immune responses with the additional dose in PLWH asymptomatic or with mild immunodeficiency. We collected peripheral blood from 72 PLWH asymptomatic or with mild immunodeficiency and from 362 non-HIV participants. The virus-neutralizing and anti-S antibodies were measured from sera, and T cell responses were measured after *ex vivo* stimulation of peripheral blood mononuclear cells (PBMCs) with the extracted whole protein of the Wuhan strain of the SARS-CoV-2 virus.

## 2 Materials and methods

### 2.1 Study design and participants

**Figure 1:**
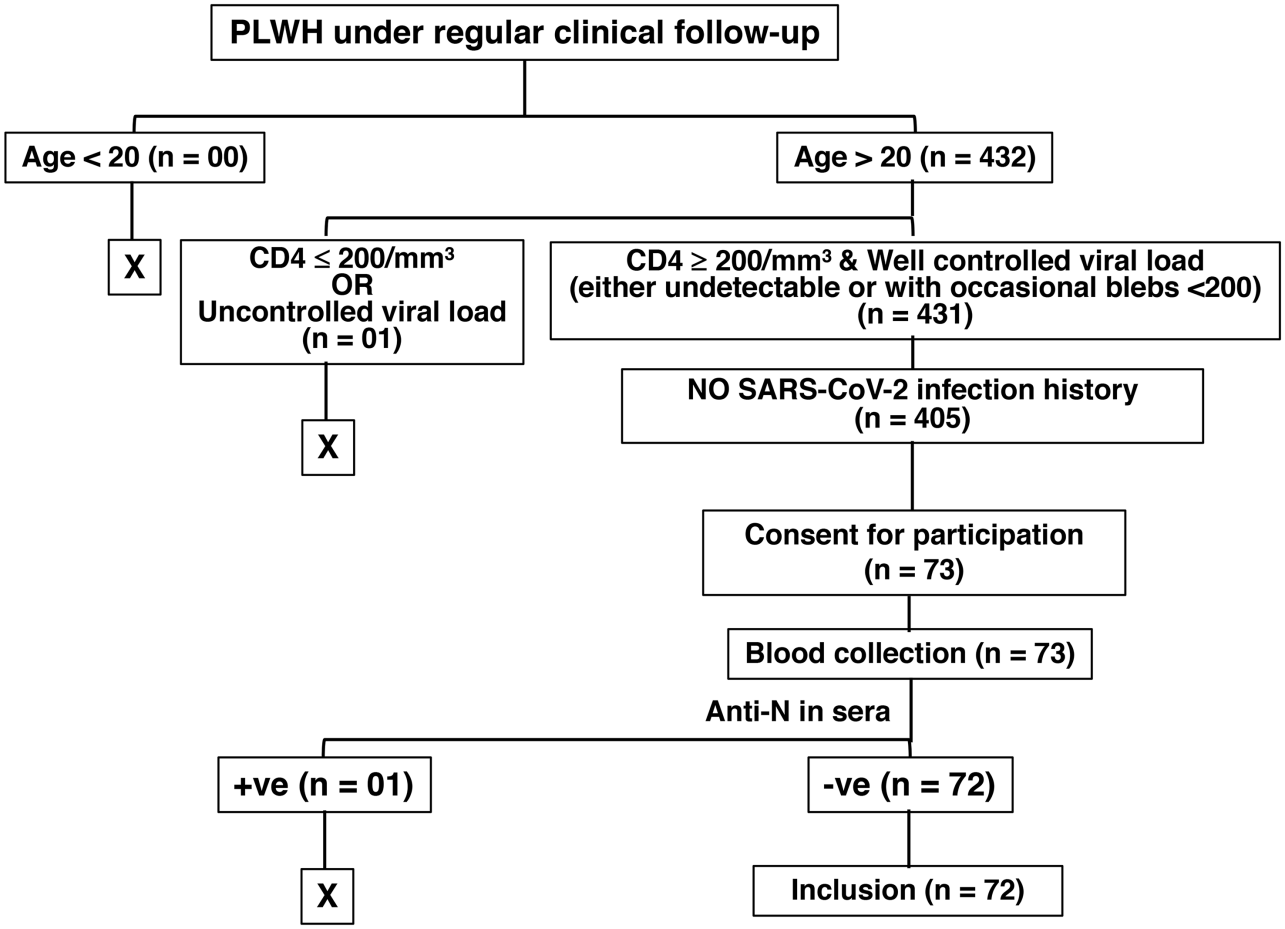
Illustration of HIV patient selection in the study. The “+ve” and the “-ve” stand for “positive” and “negative”, respectively.

We had a cohort of 431 adults (age >20 years of age) people living with HIV (PLWH) who are asymptomatic under antiretroviral treatment (ART), with CD4+ T cell counts of ≥ 200/mm^3^, no detectable HIV viral load in serum, and under routine follow-up at the Linkou campus of the Chang Gung Memorial Hospital, Taiwan. Among them, 405 PLWH had no recorded history of previous SARS-CoV2 infection. We asked all for consent to participate and only 73 of them came in to donate blood on Mondays and Wednesdays, the starting days of the experiment. One of the 73 PLWH still had anti-N antibodies in the serum and was excluded from the study. Finally, in this monocentric study, we enrolled this cohort of 72 HIV-infected individuals (PLWH, >20 years of age, 99% men, age 43 ± 10 years) whose HIV status was confirmed by a laboratory test, and the majority of them had contracted HIV through homosexuality. They are asymptomatic under ART for an average of 8 years, and minimally immunodeficient owing to their CD4+ T cell counts being ≥ 200/mm^3^ (Median = 475; Mean ± SD = 549 ± 246). We also enrolled 362 HIV-negative individuals with no history of previous SARS-CoV-2 infection (**Figure 1**). The cohort of 362 HIV-negative control individuals (25.14% men, age 44 ± 11.8 years) comprised healthcare workers (HCWs) from our hospital. Among the PLWH, 28 had received a standard two-shot regimen of either mRNA-1273 (Moderna) or ChAdOx1-S (Oxford, AstraZeneca) vaccine against SARS-CoV-2, and 44 (61.1%) had an additional shot of the mRNA-1273 (Moderna) vaccine. Among the healthcare workers without HIV infection (non-HIV), 45 had received a standard two-shot regimen, and 312 subjects (87.39%) had an additional shot. Between 3 – 4 months after the last vaccination, we collected blood from the participants from January 2022 to May 2022, before the onset of the first major COVID-19 outbreak in Taiwan. Sera were processed to evaluate serological studies, including confirmation of no previous SARS-CoV-2 infection by the definition of the absence of SARS-CoV-2 nucleocapsid (anti-N) antibodies. Fresh peripheral blood mononucleated cells (PBMCs) were stimulated with whole protein extracted from the Wuhan strain of the SARS-CoV-2 virus. The study design and experimental protocols were approved by the Chang Gung Medical Foundation Institutional Review Board (Approval# 202102398B0 and 202201499A3).

### 2.2 Quantification of total SARS-CoV-2 anti-spike and anti-nucleocapsid antibodies

We measured total antibodies (including IgG) to the SARS-CoV-2 spike protein receptor binding domain (RBD) in the sera using a commercially available Elecsys^®^ Anti-SARS-CoV-2 S kit (Cat# 09 289 275 190, Roche), as recommended by the manufacturer. We also measured antibodies (including IgG) to the SARS-CoV-2 nucleocapsid (N) protein in the sera with a double-antigen sandwich assay format using Elecsys^®^ Anti-SARS-CoV-2 kit (Cat# 09 203 079 190, Roche), as recommended by the manufacturer.

### 2.3 Quantification of SARS-CoV-2 neutralizing antibodies

The virus-neutralizing antibodies were measured through a previously described plaque reduction neutralization test, using a SARS-CoV-2 Wuhan-like variant isolated from a patient admitted at the Linkou campus of the Chang Gung Memorial Hospital, Taiwan (18). In brief, live SARS-CoV-2 virus was propagated in Vero cells in minimal essential medium (MEM) without fetal bovine serum (FBS) supplement. Serum samples were inactivated at 56°C for 30 min before use. Serial two-fold dilutions of sera were mixed with an equal volume of SARS-CoV-2 virus suspension containing 100X the median tissue culture infectious dose (TCID50). The mixture was incubated for 2 h at 37°C, and then an equal volume of suspended VeroE6 cells (approximately 30,000 cells/well) was added to each well. Following incubation for 1 week at 37°C, cells were fixed with 5% glutaraldehyde and stained with 0.1% crystal violet. Serum neutralization titers were calculated and expressed as the reciprocals of the highest serum dilution that inhibits cytopathic effects.

The quantification of virus-neutralizing antibodies was further verified with a binding inhibition assay using a MediPro SARS-CoV-2 Antibody ELISA kit. In this assay, 96-well ELISA plates were first coated with 2 μg/ml ACE2-ECD-Fc antigen in 100 μl/well coating buffer containing 0.1M sodium carbonate (pH: 9.6). The antigen was allowed to bind to the plates for overnight at 4°C, washed 6 times with 250 μl/well/wash buffer (25-fold phosphate buffer solution with 0.05% Tween 20 (pH: 7.2) using an automatic microplate washer, and extra binding sites were blocked with 200 μl/well blocking solution containing 5N HCL, Sucrose, Triton X-100, Casein, and Trizma Base. Five-fold dilutions of sera, diluted in a buffered salt solution containing carrier proteins and preservatives, were mixed with a 1:100 dilution of S1-RBD-HRP conjugate. The mixture was then incubated for 30 min at 25°C and washed. TMB substrate (3,3’,5,5’-tetramethylbenzidine diluted in citrate buffer containing hydrogen peroxide) was then added. The reaction was stopped by a diluted H_2_SO_4_ solution, and the absorbance of each well was measured at 450nm within 10 min using a microplate reader [18, MediPro SARS-CoV-2 Antibody ELISA kit (http://www2.fpg.com.tw/html/com/fbc/fbc_en/medical/products _imd_ebv_kit.html)].

### 2.4 PBMCs preparation

About 3 **ml** of the whole blood was added into a centrifuge tube containing 2 **ml** Ficoll solution (Cytiva, Taiwan), and centrifuged at 2000 rpm for 20 minutes. Without damaging the layers, PBMCs (peripheral blood mononucleated cells) were carefully isolated and resuspended into 3 **ml** RPMI. Following another round of centrifugation at 2000 rpm for 3 minutes, the cell pellets were resuspended in 1 **ml** complete RPMI with 10% FBS.

### 2.5 Preparation of the whole protein of the SARS-CoV-2 virus for *ex vivo* PBMC stimulation

The SARS-CoV-2 Wuhan-like variant was amplified using Vero E6 cells, and the culture supernatant containing the virus was mixed with a RIPA Lysis Buffer (10X; Cat# 20-188, Merck). To remove the RIPA buffer, 15 ml of the mixture was then transferred into an Amicon Ultra-15 Centrifugal Filter Unit (Cat# UFC900324, Merck), and centrifuged at 5000g for 20 min, keeping the membrane panel facing up in a fixed-angle rotor. Immediately after the centrifugation, using a side-to-side sweeping motion, a pipettor was then inserted into the bottom of the filter device to withdraw the concentrated solute containing the whole protein of the virus. The protein concentration was measured by standard protocol, and the aliquots were preserved at -80°C.

### 2.6 *Ex vivo* antigen-specific stimulation of PBMCs and evaluation of T cell functional capacity

The functional capacity of the CD4+ and CD8+ T cells was studied after *ex vivo* stimulation of PBMCs with the whole protein of the SARS-CoV-2 virus. In brief, 5 × 10^5^ PBMC in 100 μl complete RPMI medium containing 10% FBS were added to each well of U-bottom 96 well plates, 1.0 μg/ml whole protein of SARS-CoV-2 virus was then added to each well, and the total volume of the medium was adjusted to 100 μl. PBMCs of each sample without viral protein served as controls. Following antigen-specific stimulation for 4h, or for an extended period of 16h, PMA (50 ng/ml; Cat# P8139, Sigma Aldrich) and calcium ionomycin (1.0 μg/ml; Cat# I9657, Sigma Aldrich) were added with brefeldin-A (5 μg/ml; Cat# B7651, Sigma-Aldrich) to the culture to boost the stimulation for another 2h.

Following stimulation, PBMCs were centrifuged at 1800 rpm for 3 minutes at room temperature, the stimulation medium was removed, and they were surface stained with anti-CD4 (Cat# 317428, Biolegend), anti-CD8 (Cat# 555634, BD Biosciences), and anti-LAG3 (Cat# 565616, BD Biosciences) monoclonal antibodies in the dark at room temperature for 20 minutes. The surface-stained PBMCs were then washed twice with PBS, fixed with IC fixation buffer (Cat# 00822249, eBioscience) at room temperature for 15 min, and washed twice with perm buffer (Cat# 00833356, eBioscience). Intracellular cytokines were then stained with anti-IL-2 (Cat# 560707, BD Biosciences), anti-IL-10 (Cat# 564053, BD Biosciences), and anti-IFN-γ (Cat# 554702, BD Biosciences) monoclonal antibodies in the dark at room temperature for 40 minutes. Excess antibodies were then removed by washing twice with the perm buffer, and stained PBMCs were then fixed with 2% formalin. At least 100,000 lymphocytes, which includes at least 10,000 CD4+ or CD8+ T cells, were acquired through a BD LSRFortessa™ Cell Analyzer (BD Biosciences) and analyzed with FlowJo (Tree Star, Inc.) software.

In “Stimulation A”, we stimulated PBMCs with the whole viral protein retrieved from SARS-CoV-2 Wuhan-like variant and boosted the response with non-specific PMA (Phorbol-12-myristate-13-acetate) + Calcium Ionomycin re-stimulation. Side-by-side, in “Stimulation B”, PBMCs of the same donor were stimulated with PMA + Ionomycin only, as a control of non-specific activation. The values of “Stimulation A” – “Stimulation B” have been calculated for the virus-specific T cell response.

### 2.7 Statistical analysis

Data are expressed as mean ± SEM. All statistical analyses were done to compare two groups only. We used GraphPad Prism version 8 for Student’s t-test for all analyses and considered *p*-values < 0.05 as significant.

## 3 Results

### 3.1 An additional primary shot induces significantly more immunity in PLWH

**Figure 2:**
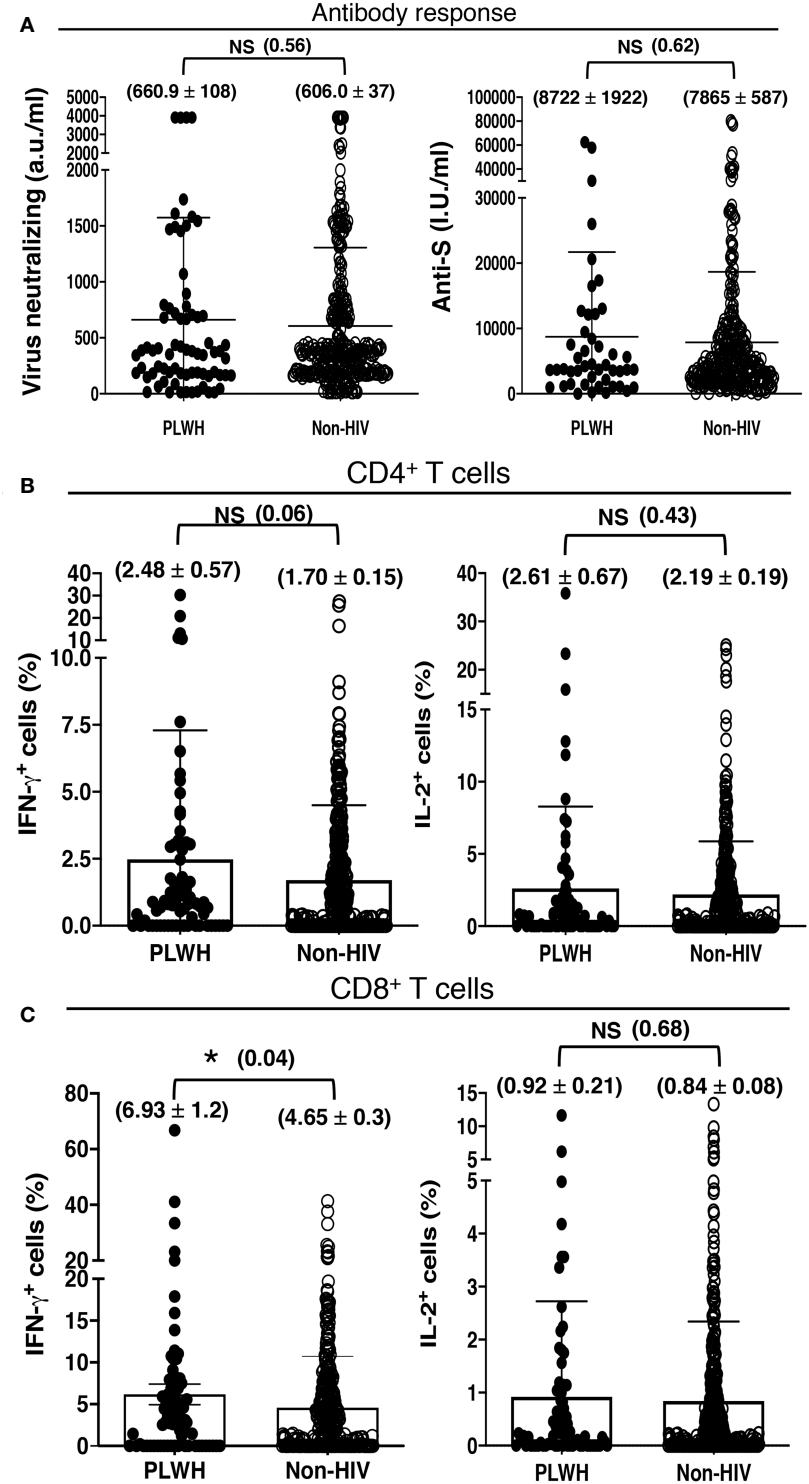
Antibody and T cell responses to COVID-19 vaccination, irrespective of the number of vaccine doses received, in 72 PLWH and 362 HIV-negative control individuals. **(A)** Serological findings following COVID-19 vaccination in PLWH and non-HIV healthcare workers. Higher, albeit insignificant, SARS-CoV-2 neutralizing and anti-Spike antibody responses to COVID-19 vaccination in our cohort of PLWH than people without HIV infection (Non-HIV). **(B, C)** T cell priming by COVID-19 vaccine in PLWH and non-HIV healthcare workers. Higher, albeit insignificant, antigen-specific **(B)** CD4+ and **(C)** CD8+ T cell responses to COVID-19 vaccination in our cohort of PLWH than people without HIV infection (Non-HIV). Each dot represents an individual, with closed circles representing PLWH. People without HIV infection (open circles) served as controls. Values are mean ± SEM, and values in parenthesis are p-values for two-tailed unpaired t-tests of comparison between the two stated populations (*=p<0.05; NS=non-significant, p>0.05).

All of our participants received the first 2 shots of either mRNA-1273 (Moderna) or ChAdOx1-S (Oxford, AstraZeneca) vaccine. They received the mRNA-1273 (Moderna) vaccine for the additional shot. The ratio of the participants receiving the mRNA-1273 (Moderna) and ChAdOx1-S (Oxford, AstraZeneca) vaccines was comparable between the PLWH and non-HIV cohorts. We found higher, albeit statistically insignificant, antibody and antigen-specific T cell response to COVID-19 vaccination in our cohort of PLWH than those without HIV infection. The PLWH cohort had 660.9 ± 108 a.u./ml virus neutralizing and 8722 ± 1922 I.U./ml anti-S antibodies, in contrast to the non-HIV cohort having 606.0 ± 37 a.u./ml virus neutralizing and 7865 ± 587 I.U./ml anti-S antibodies (**Figure 2A**). The PLWH cohort also had better T cell responses. Approximately 2.5% CD4+ T cells and 6.9% CD8+ T cells produced IFN-γ, with 2.6% CD4+ T cells and 0.9% CD8+ T cells produced IL-2 in the PLWH group. In contrast, approximately 1.7% CD4+ T cells and 4.6% CD8+ T cells produced IFN-γ, with 2.2% CD4+ T cells and 0.9% CD8+ T cells produced IL-2 in those without HIV infection (**Figures 2B, C**).

**Figure 3:**
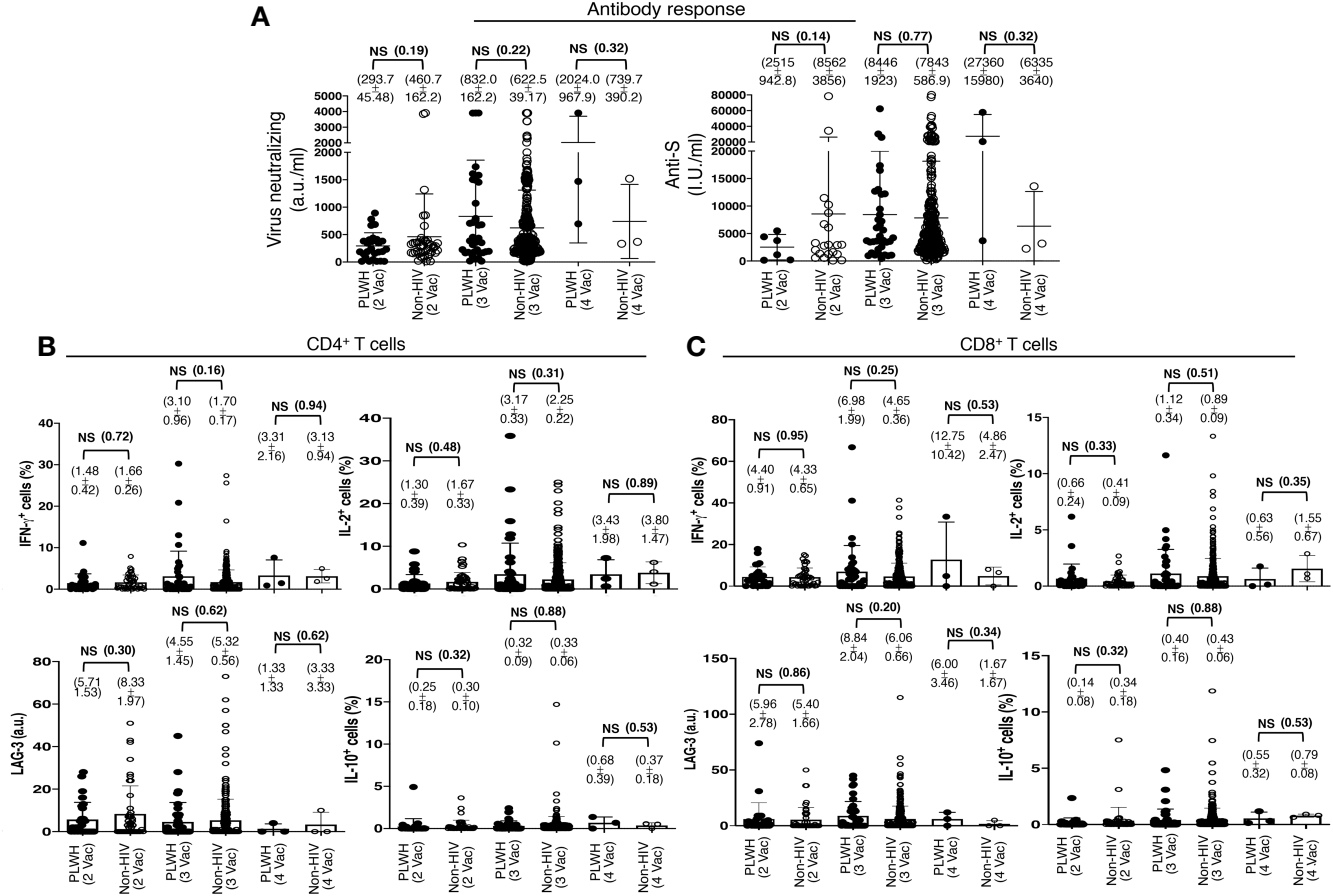
Antibody and T cell responses following the standard two-shot regimen and an additional shot of primary series COVID-19 vaccines in PLWH. **(A)** SARS-CoV-2 Virus-neutralizing and anti-spike antibodies in the sera, after the cohorts of PLWH and non-HIV subjects were grouped according to the number of COVID-19 vaccine shots they received. 2 Vac = standard two-shot regimen, 3 Vac = with an additional shot of primary vaccine series, and 4 Vac = with two additional shots of primary vaccine series. **(B, C)** SARS-CoV-2 antigen-specific T cell responses following the standard two-shot regimen and an additional shot of primary series COVID-19 vaccines in PLWH. **(B)** Effector and regulatory responses of CD4+ and **(C)** CD8+ T cells. Each dot represents an individual, with closed circles representing PLWH. People without HIV infection (open circles) served as controls. Values are mean ± SEM, and values in parenthesis are p-values for two-tailed unpaired t-tests of comparison between the two stated populations (NS=non-significant, p>0.05).

This finding was in contrast to the previous reports that showed a lesser immune response to the COVID-19 vaccination in PLWH. The search for this variation revealed that the standard two-shot regimen of the COVID-19 vaccine indeed elicited lower antibody responses in PLWH than in those without HIV infection, although the difference was statistically insignificant. The PLWH cohort had 293.7 ± 45.48 a.u./ml virus neutralizing and 2515 ± 942.8 I.U./ml anti-S antibodies, in contrast to the non-HIV cohort with 460.7 ± 162.2 a.u./ml virus neutralizing and 8562 ± 3856 I.U./ml anti-S antibodies (**Figure 3A**). The trend reversed after an additional primary shot. The PLWH cohort then had a superior antibody response than that of the non-HIV cohort, although the difference was also statistically insignificant. The PLWH cohort had 832.0 ± 162.2 a.u./ml virus neutralizing and 8446 ± 3856 I.U./ml anti-S antibodies, in contrast to the non-HIV cohort with 622.5 ± 39.2 a.u./ml virus neutralizing and 7843 ± 587 I.U./ml anti-S antibodies (**Figure 3A**).

The standard two-shot regimen of the COVID-19 vaccine elicited comparable virus-specific T cell responses in both populations. Approximately 1.5% CD4+ T cells (**Figure 3B**) and 4.5% CD8+ T cells (**Figure 3C**) produced IFN-γ, with 1.5% CD4+ T cells (**Figure 3B**) and 0.7% CD8+ T cells (**Figure 3C**) produced IL-2 in both the population, as revealed with *ex vivo* overnight stimulation of PBMC with the extracted whole protein of Wuhan strain SARS-CoV-2 virus. An additional primary shot boosted T cell responses in both populations, and a higher T cell response was measured in PLWH than in non-HIV people. In PLWH, IFN-γ production increased from 1.5% to 3% in CD4+ T cells and from 4.5% to 7% in CD8+ T cells. IL-2 production also increased from 1.3% to 3.2% in CD4+ T cells and from 0.7% to 1.1% in CD8+ T cells (**Figures 3B, C**). The expression of the induced regulatory T cell (iTreg) marker LAG-3 and the secretion of the regulatory cytokine IL-10 were not increased substantially after the third shot, both in PLWH and in the non-HIV participants (**Figures 3B, C**).

### 3.2 Higher immunity boosting with the additional primary shot in PLWH

**Figure 4:**
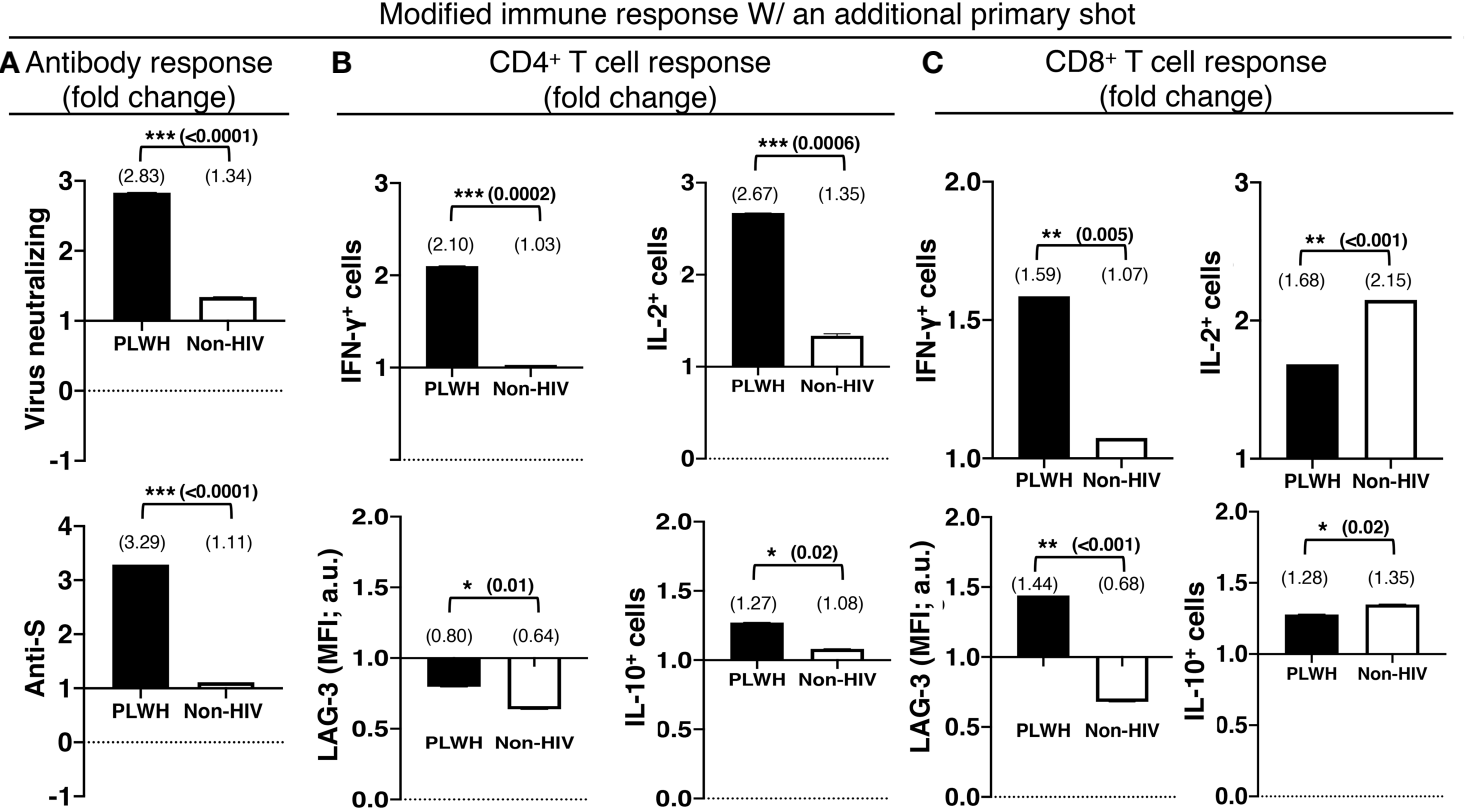
The relatively preserved functional capacity allows better immune boosting with an additional shot of the primary series of COVID-19 vaccines in PLWH. **(A)** Virus-neutralizing (upper panel) and anti-spike (lower panel) antibodies in the sera. Antigen-specific effector (upper panel) and regulatory (lower panel) responses of the **(B)** CD4+ and **(C)** CD8+ T cells. People without HIV infection (white bars) served as controls. Data are mean fold changes of stated immune responses, from the responses generated with the standard two-shot regimen to those after an additional shot (***=p≤0.0005; **=p≤0.005; *=p≤0.05; two-tailed unpaired t-test of comparison between the two stated populations).

An additional primary shot of the COVID-19 vaccine resulted in a 2.8-fold increase in virus-neutralizing and a 3.3-fold increase in anti-S antibodies in PLWH, in contrast to a mere increase of 1.3 and 1.1-fold increases in virus-neutralizing and anti-S antibodies in non-HIV people, respectively (**Figure 4A**). The boosting of T cell response with an additional primary shot was also higher in PLWH than the non-HIV population. IFN-γ-producing CD4+ and CD8+ T cells were increased by 2.1 and 1.6 fold, respectively, in PLWH, while these only increased by 1.03 and 1.07 fold in the non-HIV population (**Figures 4B, C**). The additional primary shot boosted IL-2 responses differently in PLWH and non-HIV people. IL-2-producing CD4+ T cells were increased more, 2.7 vs. 1.4 folds, and IL-2-producing CD8+ T cells were increased less, 1.7 vs. 2.2 folds, in PLWH. These augmented effector responses were accompanied by an increase in regulatory IL-10 response in both populations. In a similar trend to IL-2, IL-10 responses increased more in the CD4+ T cells and less in the CD8+ T cells of PLWH than in those of non-HIV individuals (**Figures 4B, C**). Another regulatory molecule, LAG-3, was downregulated with the additional primary shot in CD4+ T cells of both populations and in the CD8+ T cells of the non-HIV population (**Figures 4B, C**). LAG-3 expression levels were only upregulated in the CD8+ T cells of PLWH by 1.4 fold (**Figure 4B**).

### 3.3 More durable effector immune response in PLWH with an additional primary shot

**Figure 5:**
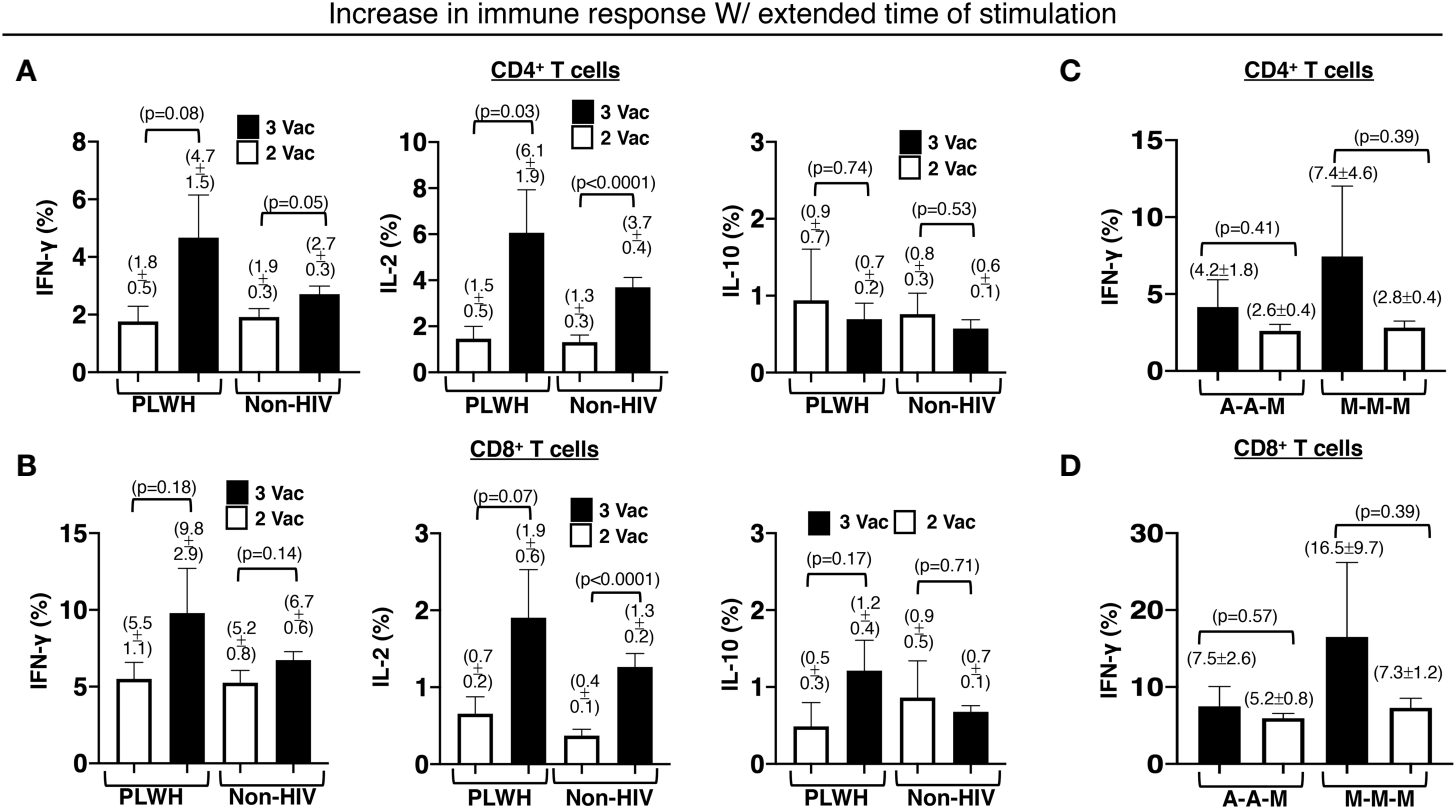
Increased and durable effector CD4+ and CD8+ T cell responses to an extended time of stimulation in PLWH. There was an increase in the stated antigen-specific responses of the **(A)** CD4+ and **(B)** CD8+ T cells, after the PBMCs were stimulated with extracted Wuhan strain SARS-CoV-2 protein for an extended period of 16h instead of a shorter duration of 4h. The same extension of the stimulation period resulted in increased and durable antigen-specific effector **(C)** CD4+ and **(D)** CD8+ T cell responses in PLWH receiving all 3 mRNA-1273 vaccines or an additional mRNA-1273 vaccine with prior 2 ChAdOx1vaccines. PBMCs from people without HIV infection served as controls. Data are mean ± SEM (p-values are of two-tailed unpaired t-tests of comparison between the two stated populations).

Relatively preserved functional capacity with the standard two-shot COVID-19 vaccine regimen facilitated higher immunity boosting with the additional primary shot in PLWH. Approximately 45% of the PLWH who received the standard two-shot regimen had the capacity to produce more IFN-γ by the T cells (46% for CD4+ and 43% for CD8+ T cells) after *ex vivo* stimulation with the extracted whole protein of the Wuhan strain of SARS-CoV-2 virus overnight than after 4 hours. The percentage of responders with similar augmented responses increased to >60% (60% for CD4+ and 63% for CD8+ T cells) for the PLWH who received an additional primary shot. In contrast, the standard two-shot regimen made a larger population of those without HIV infection (non-HIV) capable of responding more with the extended stimulation time (60% for CD4+ and 56% for CD8+ T cells). The additional primary shot failed to increase the responding population. In fact, the population shrank to 47% (45% for CD4+ and 49% for CD8+ T cells) that had higher IFN-γ response of T cells with the extended stimulation time (**Figures 5A, B**).

Within the PLWH population that responded positively with extended stimulation time, the additional primary shot also resulted in an increase in IFN-γ-producing T cells (3% increase in CD4+ and 5% increase in CD8+ T cells). Such an increase was minimal in the non-HIV population (1% increase in CD4+ and 2% increase in CD8+ T cells). A similar trend was also measured for IL-2-producing T cells (**Figures 5A, B**). In PLWH who received an additional primary shot of the mRNA-1273 vaccine, the capacity of boosting with the extended stimulation time was greater, albeit with higher variation (7.4 ± 4.6% IFN-γ-producing CD4+ and 16.5 ± 9.5% IFN-γ-producing CD8+ T cells), when they received the standard two-shot regimen of the same mRNA-1273 vaccine (**Figures 5C, D**). The additional primary shot of the mRNA-1273 vaccine, after the standard two-shot regimen of ChAdOx1vaccine, yielded a boost of 4.2 ± 1.8% IFN-γ-producing CD4+ (**Figure 5C**) and 7.5 ± 2.6% IFN-γ-producing CD8+ T cells (**Figure 5D**) in our PLWH cohort.

## 4 Discussion

At the inception of the COVID-19 pandemic, non-pharmaceutical interventions such as masking, physical distancing, and hand hygiene were the only ways to deter the infection (19–21). Vaccines, as a mock infection to coach the immune system to defend against the natural infection, were developed at an unprecedented speed and the Pfizer-BioNTech and the Moderna COVID-19 vaccines were emergently authorized within a year in 2020. There is a need for real-world evidence of vaccine effectiveness, especially with the diversity of vaccinees. We focused our research on PLWH who were asymptomatic or had mild immunodeficiency. They are under regular ART with CD4+ T cell count ≥ 200/mm^3^. The two-shot regimen was the standard for most of the vaccines to effectively prevent the infection (10–12, 22). Suboptimal immune response to the standard regimen is still a concern in immunocompromised vaccinees such as PLWH (23–25). One additional dose was recommended for an extended primary series in some countries including Taiwan.

Our results suggest a response lower in magnitude in PLWH with the two-shot regimen, although not statistically significant. Even though the two-dose regimen is of comparable immunogenicity for PLWH, the third shot elicited significantly more response in PLWH compared to people with no HIV. A plausible possibility is immune tolerance. The marginally compromised immunity in PLWH who are asymptomatic or have mild immunodeficiency deters the full-scale immune activation of two shots. This deterrence preserves the space for further activation by the third shot. However, for vaccinees without HIV, two shots achieved a higher level of activation. Immune tolerance mechanisms emerge to prevent overwhelming immune response with further activation.

However, we could not detect significant expression of regulatory markers on the T cells. Our survey of immune tolerance by checking these markers was not comprehensive. These regulatory markers have been discovered in contexts of extreme immune activation (26, 27), or in the realm of immune exhaustion (28, 29). Two vaccine shots cannot go that far, otherwise, they cannot demonstrate significant immune responses. We will work on this and try to define the subtle immune tolerance mechanism in this context. This work would help define appropriate immune activation with vaccination. People generally use Th2 cytokines for this purpose. However, Th2 cytokines are more likely due to skewing or deviation of immune activation instead of immune tolerance which regulate overshooting of the response (30).

The majority of vaccine immunity research relies on serum antibody levels only (31). Antibody responses fluctuate and are destined to decline if there is no more activation by vaccines or natural infections. Serum antibody quantification is not a good indicator of immune status. It is a critical problem to take antibodies as the immune correlate for vaccine effectiveness (32). In contrast to T cell studies, there are no standardized study protocols for B cell functional capacity such as that for T cells with *in vitro* restimulation (33). In addition, the regulatory mechanisms have only been well defined with T cells, especially in CD4+ T cells. We did T cell studies side by side with the B cell antibody responses. There is rarely research of this kind. We expect more studies dissecting T cell responses or even the functionalities of B cells will make vaccine research advance further.

Immune responses are very heterogenous among individuals, with huge variations either in antibody levels or T cell responses (34, 35). We saw a trend of difference by mean values, and the significance of this trend could only be verified with a large number of study participants. This heterogenicity and the limited number of participants are the major limitations of our study. However, our study results are relatively well-defined and evidently imply the possible role of immune tolerance with repeated shots of vaccination. This is a concern when designing vaccination programs for optimized immunity to prevent infection, severe disease, or death.

## Data availability statement

The original contributions presented in the study are included in the article/supplementary material. Further inquiries can be directed to the corresponding authors.

## Ethics statement

The studies involving humans were approved by Chang Gung Medical Foundation Institutional Review Board (Approval# 202102398B0). The studies were conducted in accordance with the local legislation and institutional requirements. The participants provided their written informed consent to participate in this study.

## Author contributions

AD and C-TH designed the research studies, analyzed the data, and wrote the paper; C-YH, S-HH, C-GH, C-SC, Y-LH, and G-YC performed the research. All authors contributed to the article and approved the submitted version.
